# Serum Behavior of NT-3 and VEGFβ, Two Unstudied Growth Factors in Patients with Diabetes Mellitus and End-Stage Renal Disease

**DOI:** 10.3390/jcm14217585

**Published:** 2025-10-25

**Authors:** Mihaela Gheorghiu, Maria-Florina Trandafir, Octavian Savu, Daniela Pasarica, Coralia Bleotu

**Affiliations:** 1 Pathophysiology and Immunology Department, “Carol Davila” University of Medicine and Pharmacy, 020021 Bucharest, Romania; 2 “N.C. Paulescu” National Institute of Diabetes, Nutrition and Metabolic Diseases, 011233 Bucharest, Romania; octavian.savu@umfcd.ro; 3 Doctoral School, “Carol Davila” University of Medicine and Pharmacy, 020021 Bucharest, Romania; 4 “Stefan S. Nicolau” Institute of Virology, 030304 Bucharest, Romania

**Keywords:** end-stage renal disease, diabetes mellitus, neurotrophin-3, vascular endothelial growth factor beta, interleukin-10

## Abstract

**Background.** DM frequently causes ESRD, a situation in which survival is impossible without chronic dialysis. This research aimed to study the tissue-regenerative capacity of ESRD patients with/without DM. For this purpose, two growth factors were studied: NT-3 and VEGFβ. To our knowledge, this is the first study on the serum behavior of NT-3 in patients with ESRD + DM and, very likely, in patients with ESRD alone. VEGFβ is also very little studied in these patient categories. Since the quasi-permanent inflammation was already clearly proven, this study focused on the anti-inflammatory capacity, taking IL-10 as a prototype, and also the interrelationships between these three factors. **Method.** All the aforementioned compounds were determined from serum samples, utilizing ELISA kits. **Results.** The results were surprising, proving a marked polarization of their serum behavior. Although the mean serum levels of NT-3 and VEGFβ were significantly increased in patients with ESRD + DM compared to those with ESRD alone, most patients in both groups had serum levels below the detection limits of the kits used. **Conclusions.** Although this research is in its early stages, it generates important conclusions and directions for further research, as the functional connections between the studied parameters are not fully understood.

## 1. Introduction

Diabetes mellitus (DM) and chronic kidney disease (CKD) are two pathologies that are frequently associated and mutually potentiate each other. It is known that, at present, diabetes mellitus (type 1 and type 2) and hypertension are the most common causes of CKD, and therefore of ESRD (end-stage renal disease) [1,2,3]. Statistical data suggest that in 2021, 11% of the general population had DM (approximately 537 million people). Projections at that time suggested that by 2025, the number of individuals with DM would reach 783 million [4]. It is also known that 30–40% of patients with DM develop diabetic kidney disease (DKD), the most common cause of ESRD [5,6,7]. This is a situation in which most patients require chronic dialysis or kidney transplantation.

To understand all the data presented in this paper, it is necessary to know the pathophysiological mechanisms that act and interact in patients with DM and ESRD. DM generates strongly interconnected hemodynamic, metabolic, and inflammatory changes at the renal level. To present them correctly, the discussion must start with the renal morphofunctional unit, the nephron, composed of the renal corpuscle (glomerulus and Bowman’s capsule), and the renal tubule (proximal convoluted tubule, loop of Henle, and distal convoluted tubule). Several distal convoluted tubules empty into a collecting duct.

The most important mechanism induced by DM is the glomerulotubular structural and functional alteration, which consequently leads to mesangial proliferation, glomerular basement membranes (GBMs) thickening, glomerulosclerosis, and interstitial fibrosis [8].

At the molecular level, the pathophysiological mechanisms include metabolic and hemodynamic changes, innate and acquired immune reactions accompanied by fibrosis, and the intervention of growth factors.

**Metabolic Changes**

They are generated both directly by hyperglycemia (a) and by the products of glucose metabolism (b).

- Prolonged hyperglycemia induces glycation of proteins (including collagen), lipids, and the extracellular matrix [9]. This results in the formation of AGEs (advanced glycation end products), which generate receptor-mediated and non-receptor-mediated damage. AGEs activate RAGEs (receptors for advanced glycation end products), triggering signaling cascades that induce excessive production of ROS (reactive oxygen species) and activation of the transcription factor NF-kB [9,10]. Consequences: hypertrophy of renal cells incapable of mitosis, angiogenesis, ECM (extracellular matrix) synthesis, and inflammation induced and maintained by ROS that alters the structure and function of endothelial cells.

AGEs also act in a non-receptor-mediated manner both inside and outside cells. In cells, they induce a decrease in NO (nitric oxide) bioavailability. Outside the cells, glycated collagen can attach to various molecules. Consequences: connective tissue structure and function alteration, GBM thickening, and decreased protein catabolism, with a consequent increase in the interstitium and mesangial matrix volume [9,10,11].

b. Altered glucose metabolism induces the development of diabetic complications. Three biochemical pathways are most involved: polyols, hexosamine, and protein kinase C (PKC).

*Polyol pathway*. Increased intracellular glucose activates aldose reductase, producing sorbitol, which is then converted to fructose by sorbitol dehydrogenase. Aldose reductase uses a proton donated by NADPH (nicotinamide adenine dinucleotide phosphate). Thus, the protons are consumed and can no longer neutralize ROS. Not all tissues have sorbitol dehydrogenase. The retina, kidneys, and Schwann cells do not contain it, so in diabetic patients, sorbitol accumulates in toxic concentrations and causes complications in these organs [12,13].

*Hexosamine pathway*. Glycoproteins, proteoglycans, glycolipids, and glycosaminoglycans are physiologically synthesized by this pathway [14,15]. The key enzyme is GFAT (glutamine-fructose-6-phosphate-aminotransferase). But this enzyme also stimulates the gene expression of TGFβ1 (transforming growth factor beta 1), a cytokine involved in the development of diabetic nephropathy [16]. The main cause of the development of renal failure in patients with DM1 and DM2 has been shown to be the stimulation by TGFβ1 of the laminin, fibronectin, and collagen overproduction, with an increase in the mesangial matrix [17,18,19,20,21].

*PKC pathway*. In DM, in addition to the physiological activation pathway involving phospholipase C (PLC), inositol triphosphate (IP3), diacylglycerol (DAG), massive release of Ca2+ into the cytosol, and the possible presence of phosphatidylserine [22], mechanisms induced by prolonged hyperglycemia are also added. Thus, excessive glycolysis is activated and generatesincreases in the cytosolic levels of glyceraldehyde-3-phosphate, and glycerol-3-phosphate. These increases result in theactivation of DAG synthesis and, indirectly, PKC. In DM, PKC can also be activated by ROS and AGEs [9]. Consequences: vasoconstriction, Na+ reabsorption in the proximal convoluted tubules, platelet aggregation, positive inotropic effect, stimulation of gluconeogenesis and glycogenolysis (thus maintaining hyperglycemia), bronchoconstriction, and the activation of T and B cells [22,23].

**Hemodynamic Changes**

In diabetes, glomerular filtration increases by decreasing the tone of the afferent arteriole and increasing the tone of the efferent one; however, the causes are not very clear. There are two theories that try to justify these changes. The first supports the intervention of two categories of circulating molecules: vasoconstrictors of the efferent arteriole (angiotensin II—AG II, thromboxane A2—TXA2, endothelin-1—ET-1, and ROS) and vasodilators of the afferent one (NO, prostaglandins—PG, the kinin-kallikrein system, atrial natriuretic peptide—ANP, and insulin) [24,25,26]. The second theory claims that glomerular hyperfiltration is induced by tubular mechanisms: hyperglycemia determines increased glucose in the glomerular filtrate and proximal convoluted tubule that, in turn, generatesincreased reabsorption of glucose and sodium at this level. In these conditions, adecreased level of sodium reaches the distal tubule and induces tubuloglomerular feedback (dilation of the afferent arteriole and constriction of the efferent one). Added to this is the action of insulin, which increases glucose and sodium reabsorption in the proximal tubule, but can also directly induce vasodilation of the afferent arteriole (NO-mediated action) [27].

**The innate and adaptive immune reactions accompanied by fibrosis**

Both the specialized scientific literature and our studies [28] have demonstrated the presence of complex immunological alterations in DM and ESRD, acting both as causal factors and as accompanying phenomena. It is certain that the activation of innate immunity (inflammation), as well as the periodic activation of acquired immunity (humoral and cellular immune responses), are produced by DM, ESRD, and other causes. Humoral and cellular immune responses are not effective unless they are accompanied by inflammation that completes the defense reaction. Renal inflammation is generically activated by PAMPs (pathogen-associated molecular patterns) and DAMPs (damage-associated molecular patterns). The latter are caused by membrane alterations or by the release of modified metabolic compounds from endothelial cells, tubular nephrocytes, podocytes, and mesangial cells. PAMPs and DAMPs activate TLR (toll-like receptors) and NOD (nucleotide-binding oligomerization domain-like receptors) of KRMs (kidney-resident macrophages) [29]. KRMs play an essential role in both the defense and the development of renal pathology. They function in conjunction with macrophages derived from blood monocytes (also called infiltrating macrophages) [30]. Like other tissue-resident macrophages, KRMs not only have an immunological role, but also participate in the regulation of renal function. They are activated in the M1 phenotype by ROS, AG II, and mineralocorticoids [31]. The M1 phenotype participates in the inflammatory reaction and cooperates with TH1 lymphocytes, activating cellular immunity [32,33]. The result is the structural and functional alteration of endothelial cells, tubular nephrocytes, podocytes, and mesangial cells. When the PAMPs and DAMPs numbers decrease, KRMs begin to secrete cytokines (especially TGFβ1) that stimulate fibroblast proliferation and fibrosis, as well as the expansion of the mesangial matrix, by inhibiting metalloproteinases [31,33]. We can conclude that the main actor in the occurrence of renal failure generated by chronic infections and DM is represented by KRMs, which, in addition to inflammation, activate different types of lymphocytes and induce the aforementioned structural and functional changes.

**Growth factor intervention**

They are secreted at the end of each inflammatory reaction for the restoration of destroyed parenchymal cells, vessels, nerve endings, and connective tissue. The difficulty arises when inflammation lasts a long time, with many simultaneous foci. Another problem is the ability of renal cells to enter the cell cycle and mitosis. Most of them, especially those in the proximal tubules, do not divide, having a very low physiological turnover rate. Most renal cells are arrested in the G0 phase of the cell cycle [34]. In ESRD, associated or not with DM, the involvement of TGFβ, VEGF (vascular endothelial growth factor), IGF-1 (insulin-like growth factor 1), and CTGF (connective tissue growth factor) is clearly demonstrated [9,35,36,37]. All these growth factors stimulate the entry into the cell cycle of all types of renal cells, triggering apoptosis in those that cannot divide and the appearance of fibrosis.

Conclusion: The general characteristic of ESRD is the presence of growth factor-induced renal fibrosis, involving both fibroblasts and tubular nephrocytes, podocytes, and mesangial cells [38,39].

## 2. Purpose of Study

The results we present in this article are part of an extensive study on the intestinal microbiota and immunological characteristics of patients with DM and ESRD, the general aspects of which have already been published [28]. However, we consider it very important to discuss in more detail the behavior and functional interferences between two growth factors, neurotrophin-3 (NT-3) and VEGFβ, and the influence of the post-inflammatory phase of tissue regeneration, represented by IL-10.

What are the reasons?

1. It is known that the two pathologies, DM and ESRD, are characterized by a significant/severe decrease in tissue-regeneration capacity. We will try to quantify this change by studying the two growth factors, NT-3 and VEGFβ.
2. Why NT-3?

Because DM is associated with diabetic neuropathy changes. On the other hand, ESRD is associated with uremic neuropathy changes. Which type of neuropathy predominates?

Studies conducted in this category of patients focused on BDNF (brain-derived nerve factor) and NGF (nerve growth factor). After reviewing the specialized literature, we can say that this is the first study conducted on NT-3 in both patients with ESRD + DM and those with ESRD only. Because it has different receptor-binding characteristics and targets different neuronal populations than BDNF and NGF, we considered it important and useful to conduct this study.

3. Why VEGFβ?

Like NT-3, VEGFβ is unique from VEGFα and total VEGF, which have been studied more extensively. The difference stems from both more restrictive receptor binding and the lack of direct stimulation of angiogenesis. In contrast to VEGFα, VEGFβ supports vascular metabolism, stimulates coronary vessel growth, and protects cells against mitochondrial dysfunction (a feature seen in ischemic heart disease and neurological disorders). What happens to VEGFβ in the kidneys in DM ± ESRD? What is the impact of these pathologies on VEGFβ behavior in the rest of the body’s vessels?

Another important argument is that this is also one of the extremely few studies which have been conducted only on VEGFβ (not total VEGF or VEGFα) in these categories of patients.

4. Why IL-10?

IL-10 is a cytokine specific to the late period of inflammation, having, classically, anti-inflammatory action. Its actions are, however, much more complex. At the end of inflammation, regeneration and fibrosis phenomena begin, and it has been proven that during the same period the synthesis of growth factors also increases. It is obvious that there may be a correlation between IL-10 and the behavior of NT-3 and VEGFB. Should we not highlight it?

5. We believe that this study can bring improvements in the understanding of the very complex pathophysiological mechanisms in ESRD, associated or not with DM.
6. Furthermore, it could lead to the identification of new prognostic and therapeutic targets and a deeper understanding of the exchange of biological information between growth factors.

## 3. Patients and Methods

### 3.1. Patients and Serum Markers

The patients were included in the study between September 2023 and March 2024, randomly, provided they met the inclusion and exclusion criteria. Sample taking and evaluations were performed as the patients came for their scheduled visits at specialized dialysis centers.

*Inclusion criteria*:

- patients with ESRD, some of them with associated DM2;
- undergoing hemodialysis, receiving treatment three times a week.

*Exclusion criteria*:

- acute inflammatory and infectious diseases;
- current immunosuppressing treatment;
- acute organ failure (cardiac and hepatic);
- malignancies;
- acute vascular disease (stroke and myocardial infarction);
- psychiatric conditions or impaired judgment;
- blood transfusions in the last 3 months;
- hemoglobinopathy or anemia from other causes than CKD.

Regarding these inclusion and exclusion criteria, two groups of hemodialysis patients were established to be included in the study: one group consisting of 15 diabetic patients with end-stage renal disease (DM group) (9 men and 6 women, with an average age of 63 ± 11.8 years) and another, used as a control group, consisting of 45 hemodialysis patients without diabetes mellitus (non-DM group) (33 men and 12 women, with an average age of 61.5 ± 15.1 years). The ratio of the DM to non-DM group is approximately 1 to 3. The patients in the hemodialysis diabetic group had a CKD duration between 1 and 14 years, while the non-diabetic hemodialysis patients had a CKD duration between 1 and 24 years. The average age of dialysis in non-DM patients is 5.29 ± 4.71 years, while in diabetic patients it is 2.53 ± 2.69 years. The results are systematized in the table below (Table 1).

### 3.2. Method

Venous blood samples were collected in the morning, before other investigations, from the patients following overnight fasting. The samples were aliquoted and stored at −80 °C until selected assays were performed. At the time of collection, no patients showed clinical signs of inflammation.

All the aforementioned compounds were determined from serum samples, utilizing the Merck Millipore ELISA kits for IL-10, NT-3, and VEGF β.

The human ELISA kits utilized in our study were single-wash 90 min sandwich ELISA designed for the quantitative measurement of human serum, plasma, and cell culture supernatant. The SimpleStep ELISA^®^ technology employs capture antibodies conjugated to an affinity tag that is recognized by the monoclonal antibody used to coat the SimpleStep ELISA^®^ plates. This approach to sandwich ELISA allows the formation of the antibody–analyte sandwich complex in a single step, significantly reducing assay time.

### 3.3. Statistical Analysis

Data was processed using the SPSS IBM V26 statistical package with a statistical significance defined as *p* ≤ 0.05, conventionally adopted for checking the degree of data compatibility with the null hypothesis. The statistical protocol was applied according to the design of a cross-sectional study and the type of data collected. Descriptive statistics, such as mean ± standard deviation, median, and range, were used for quantitative variables presentation (such as age, duration of hemodialysis, and serum markers). For comparison between groups (DM patients vs. non-DM patients, etc.), the “Independent—Samples *t* Test” or nonparametric tests (Independent—Samples Mann–Whitney U Test and Kruskal–Wallis Test) were applied after data normality distribution was verified. Qualitative variables (such as gender and the presence or absence of DM) have been described as structure or intensity indicators (absolute or relative frequencies). Chi Square-X^2^ or Fisher’s Exact Test were also applied to examine the association between the categorical variables. Correlation analysis was also performed. Box-plot graphics (the minimum, first quartile, median, third quartile, the maximum, and the outliers of the parameters data set) were drawn to show the data series distribution for global sample or stratified into subgroups of interest.

In statistical interpretations, the role of sample size in data accuracy is also recognized. The Romanian Dialysis Guide from 2023 informs us about the existence of 200 dialysis patients/1 million individuals in the general population [40]. Considering that Bucharest has approx. 2 million inhabitants, the number of dialysis patients is approx. 400. The sample size used (63 patients out of 400, i.e., approx. 16%) is considered to be absolutely representative of any dialysis patient in a clinic in Bucharest of the same equipment and performance as the one in the study.

It is important to note that the purpose of this paper is not to discuss the level of significance, as per the proposal of ASA; we do not believe that an effect exists just because it was statistically significant! However, according to our purpose and to the important discoveries of our study, we consider that the classic convention of *p*-value less or equal to 0.05 is a sufficient orientated measure that helps us to develop our research protocol.

### 3.4. Ethical Considerations

This study was approved by the Ethics Commission of “N.C. Paulescu” National Institute of Diabetes, Nutrition and Metabolic Diseases (Bucharest, Romania; approval no. Certif.5911/4 October 2019). All participants gave their written informed consent upon inclusion in the study. The study was conducted in accordance with the Declaration of Helsinki and was approved through Ethical Permit no. 295/17 January 2023.

## 4. Results

Both DM and non-DM groups were comparable regarding the possible confounding factors; there were no significant differences (*p* > 0.05) in age, gender distribution, duration of hemodialysis, or comorbidities between the DM and non-DM groups.

An initial evaluation of the measurements performed showcases an extremely high level of inflammation in hemodialysis patients [28]. Therefore, all serum values of growth factors and IL-10 must be interpreted in the context of the strong quasi-permanent inflammation in both groups of ESRD patients.

### 4.1. NT-3

**Figure 1:**
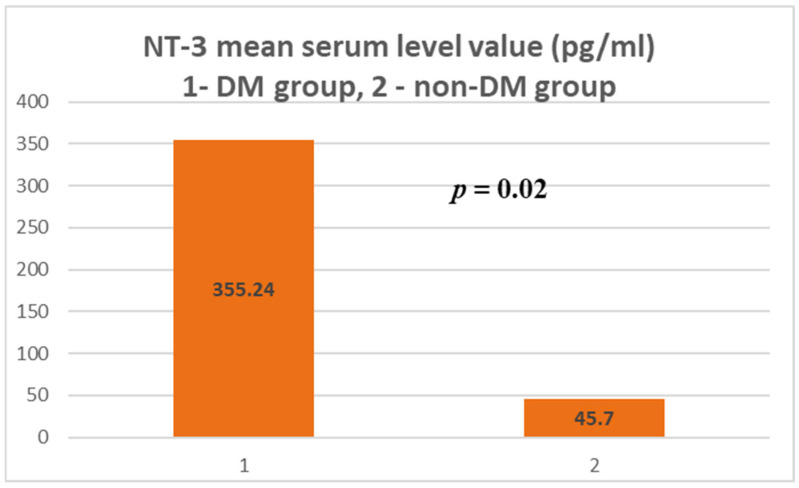
NT-3 mean serum level.

**Figure 2:**
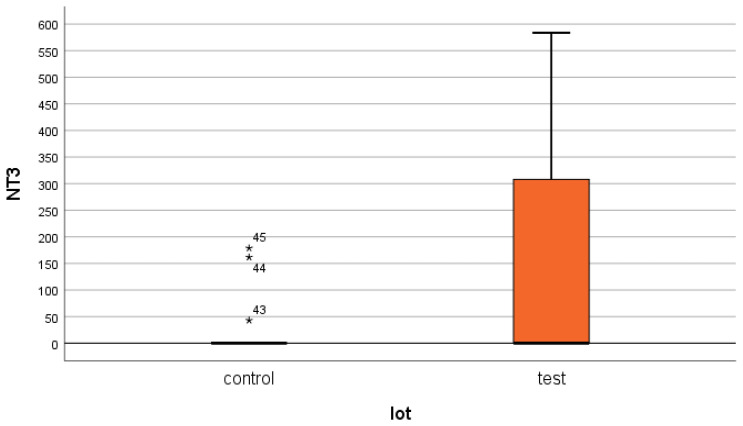
NT-3 mean serum level with standard deviation (control: non-DM and test: DM). The asterisks represent the outlier values.

For NT-3, a serum concentration of 354.24 ± 916.64 pg/mL was obtained in the test group (ESRD + DM), compared to 45.7 ± 254.64 pg/mL in the control group (non-DM) (Figure 1 and Figure 2). The difference is statistically significant (*p* = 0.02).

Thus, the serum level of NT-3 in hemodialysis patients with DM is 7.8 times higher than in those without DM. Comparing the values obtained in the two groups to the internationally accepted upper limit of normal (5.74–20 pg/mL) [41], values 15.26 times higher in the test group and 2.28 times higher in the non-DM control group are found. The maximum serum level of NT-3 in the test group was 3594.43 pg/mL, compared to 1718.71 pg/mL in the control group (two times higher).

**Figure 3:**
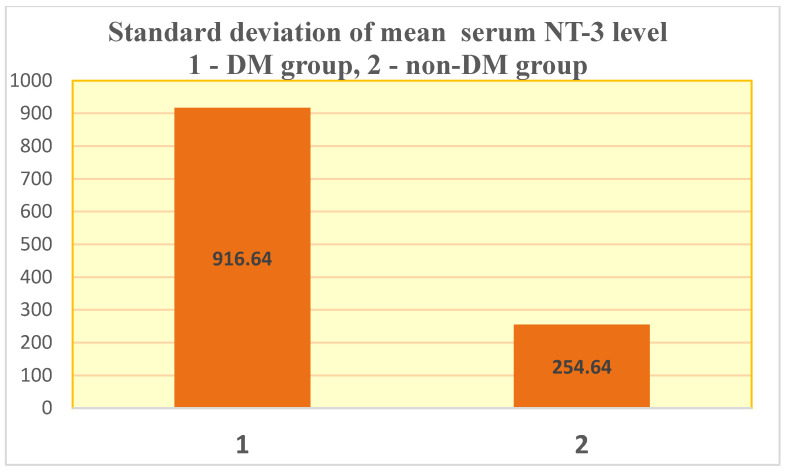
Standard deviation of mean serum NT-3 level.

What surprised us the most was the very inhomogeneous behavior of NT-3 in both the studied patient groups (Figure 2). Thus, the standard deviations were very large (Figure 2 and Figure 3).

**Figure 4:**
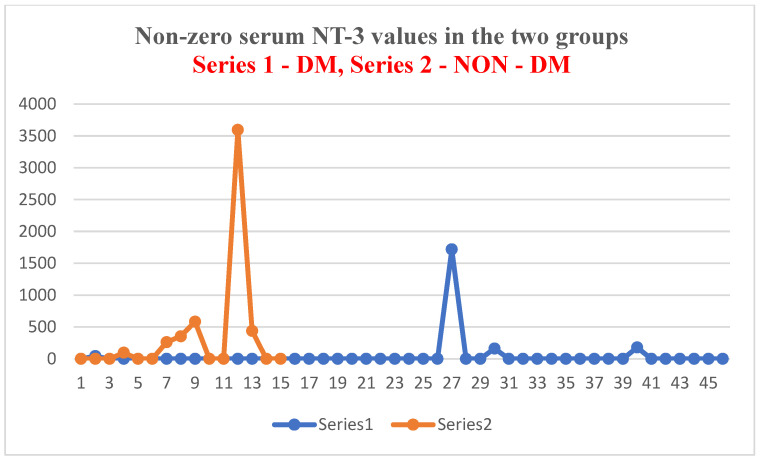
Non-zero serum NT-3 values in the two groups.

**Figure 5:**
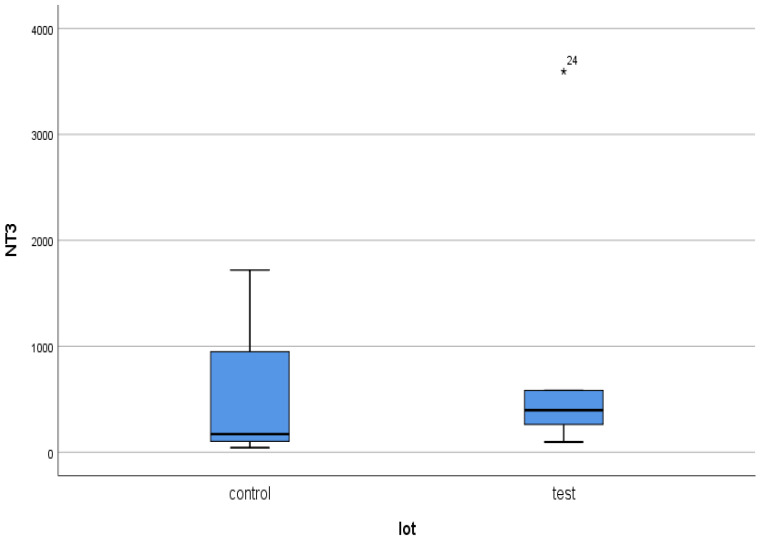
Box and whisker graphs for those patients with non-zero serum NT-3 values (control: non-DM and test: DM). The asterisk represents outlier values.

In other words, of the 15 patients in the test group (ESRD + DM), 6 patients had non-zero serum NT-3 levels (the minimum detectable dose of human NT-3 of the ELISA kit used is 4 pg/mL). This means that 40% of patients had a serum NT-3 level above 4 pg/mL. Of the 48 patients in the test group (ESRD without DM), only 4 had non-zero serum NT-3 levels (only 8% had NT-3 levels above 4 pg/mL) (Figure 4 and Figure 5).

From the beginning, we noticed a dual behavior in patients with the same diagnosis: some of them have very high serum concentrations of NT-3, while others have an inhibited synthesis capacity for this growth factor. What could be the explanation?

### 4.2. VEGFβ

**Figure 6:**
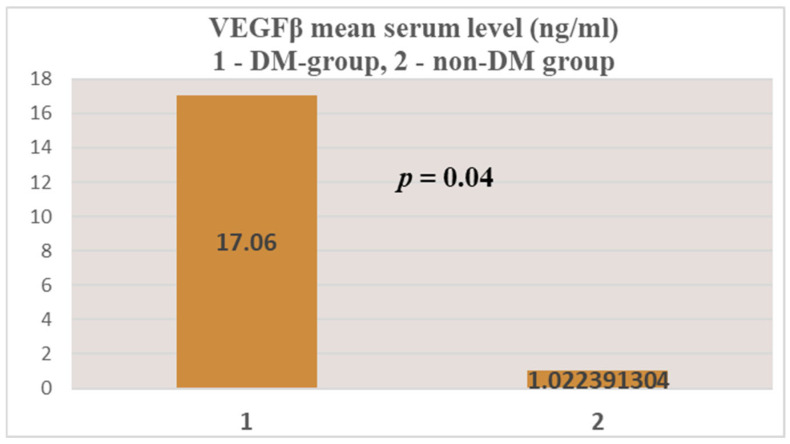
VEGFβ mean serum level.

**Figure 7:**
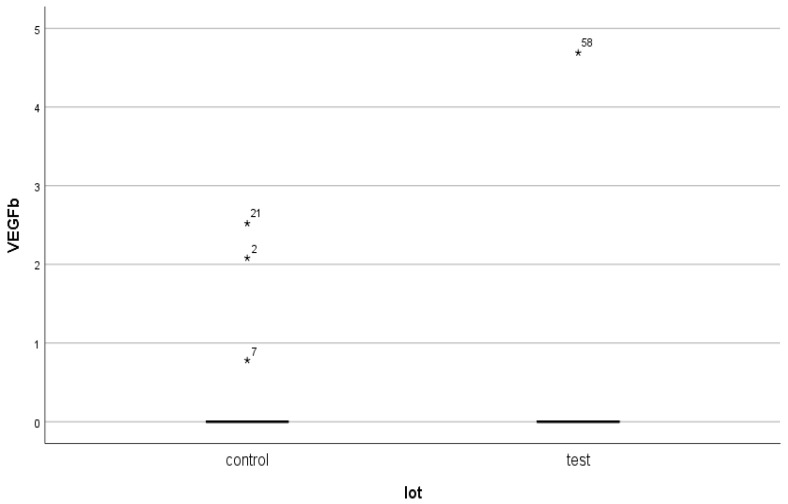
VEGFβ mean serum level with standard deviation (control: non-DM and test: DM). The asterisks represent outlier values.

For VEGFβ, in the test group (group 1—DM) an average serum level of 17.06 ± 64.8 ng/mL was obtained, compared to 1.02 ± 6.14 ng/mL in the control group (group 2—non-DM) (Figure 6 and Figure 7). As with NT-3, the difference between the two groups was statistically significant for this growth factor (*p* = 0.04).

Mean serum VEGFβ levels were 17-fold higher in patients with ESRD + DM compared to those without DM. The maximum values were 251.21 ng/mL in the test group, compared to 41.65 ng/mL in the control group (6 times higher). When comparing this with the serum VEGFβ levels considered normal at the international level (24.73–467.7 pg/mL) [42], we observe that patients with ESRD + DM have an average serum level over 36 times higher than the upper limit of normal, while in patients with ESRD alone it is 2 times higher.

**Figure 8:**
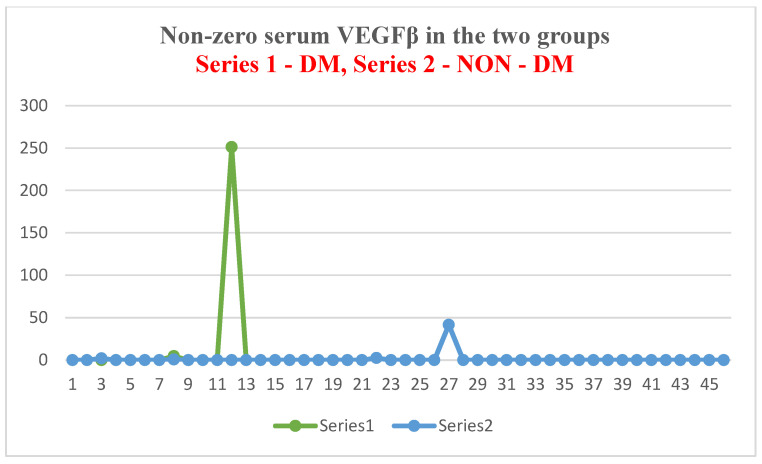
Non-zero serum VEGFβ values in the two groups.

**Figure 9:**
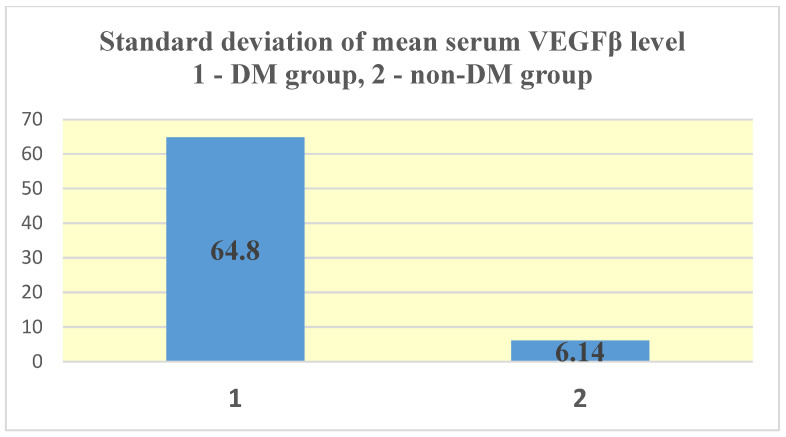
Standard deviation of mean serum VEGFβ level.

**Figure 10:**
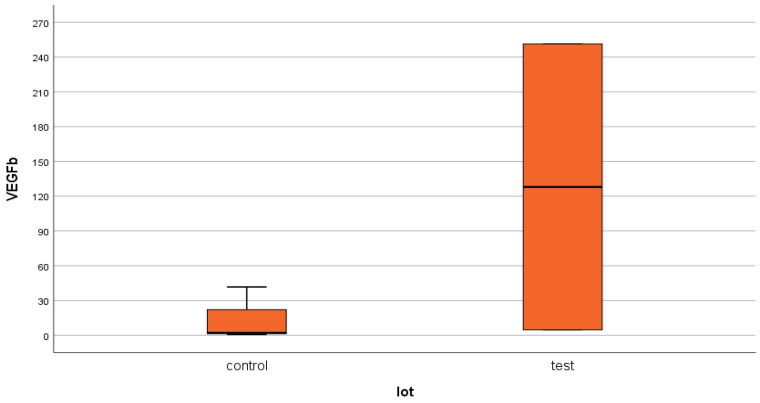
Box and whisker graphs for those with non-zero serum VEGFβ values (control: non-DM and test: DM).

As in the case of NT-3, we found a very high level of inhomogeneity in the serum levels for VEGFβ, which generated a large standard deviation of the mean (Figure 7, Figure 8 and Figure 9). Thus, of the 15 patients in the test group, only 2 (13.33%) presented non-zero serum values of VEGFβ (meaning values above 0.4 ng/mL), while of the 48 patients in the control group, 4 had non-zero values (8.33%). The rest showed values below the detection limit of the ELISA kit used (0.4 ng/mL, that is, 400 pg/mL) (Figure 8 and Figure 10). Taking into account that the serum VEGFβ values considered normal are somehow at the detection limit of the kit used, we can conclude that the rest of the patients in the two groups present normal or low values.

### 4.3. IL-10

**Figure 11:**
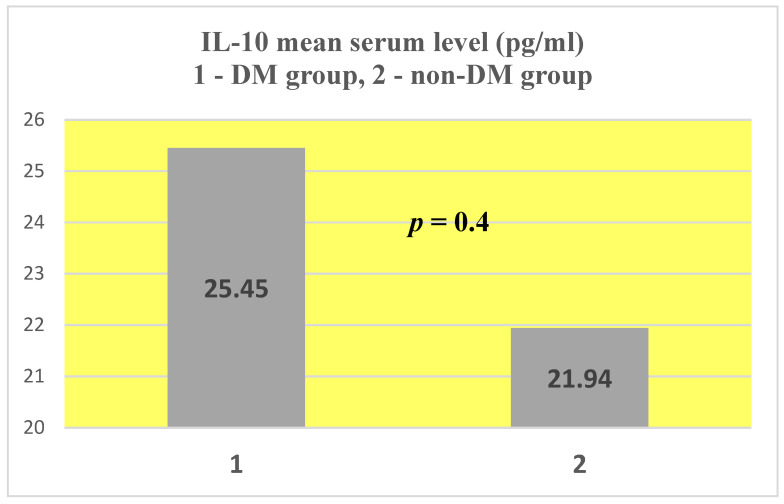
IL-10 mean serum level.

**Figure 12:**
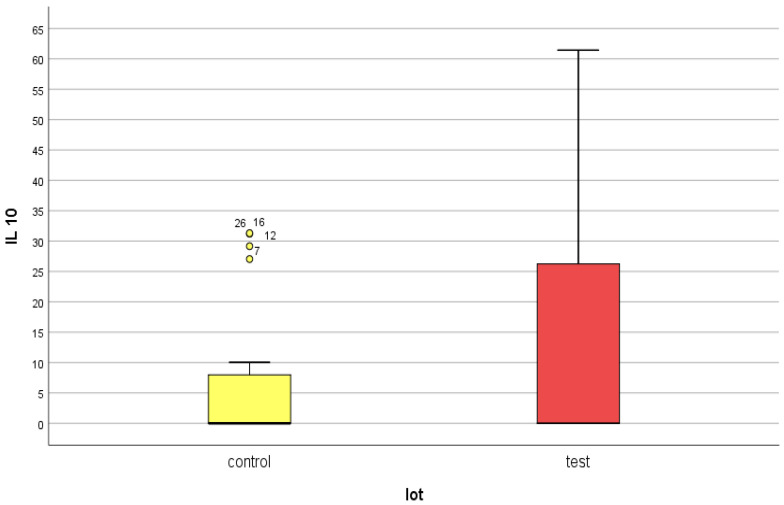
IL-10 mean serum level with standard deviation (control: non-DM and test: DM). The outlier values are represented by circles/dots.

For IL-10, a mean serum level of 25.45 ± 61.83 pg/mL was obtained in the test group (ESRD + DM) and 21.99 ± 57.18 pg/mL in the control group (ESRD non-DM) (Figure 11 and Figure 12). The difference between the two groups did not show statistical significance (*p* = 0.4).

Comparing the values obtained with the internationally accepted normal serum level of IL-10 (4.8–9.8 pg/mL), we observe an increase in both the groups studied, with values over double the upper limit of normal (2.6 and 2.2 times higher, respectively).

The maximum levels obtained were very close: 239.32 pg/mL in the test group compared to 238.3 pg/mL in the control group, both over 20 times higher than the upper limit of normal.

However, it should not be forgotten that these very high values of the anti-inflammatory marker IL-10 are present against the background of proven strong inflammation [28].

**Figure 13:**
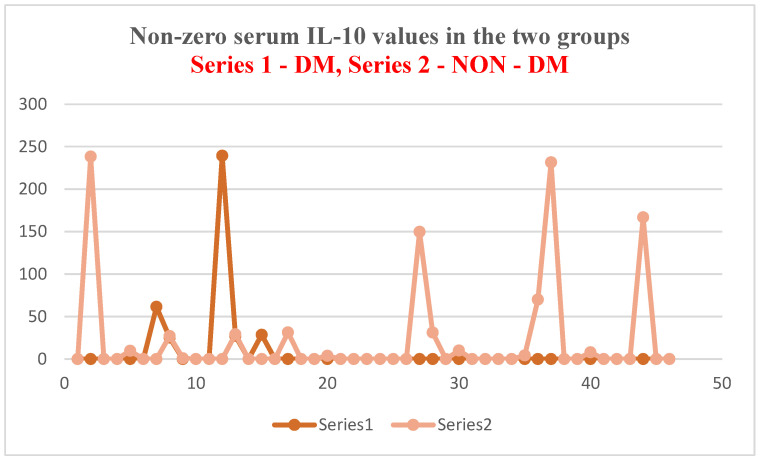
Non-zero serum IL-10 values in the two groups.

**Figure 14:**
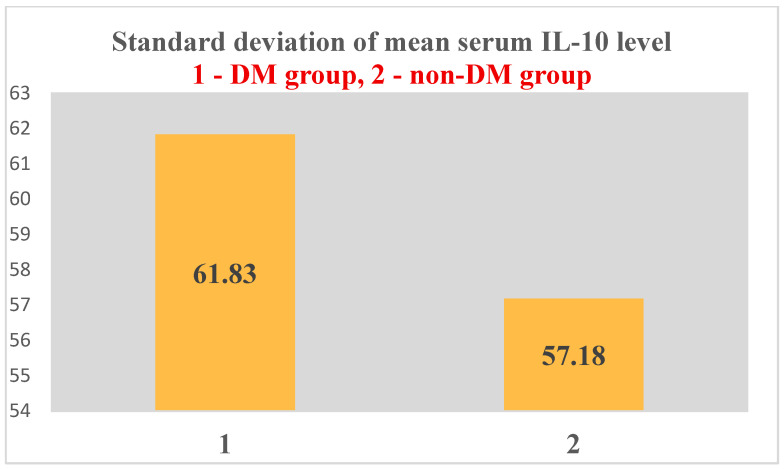
Standard deviation of mean serum IL-10 level.

**Figure 15:**
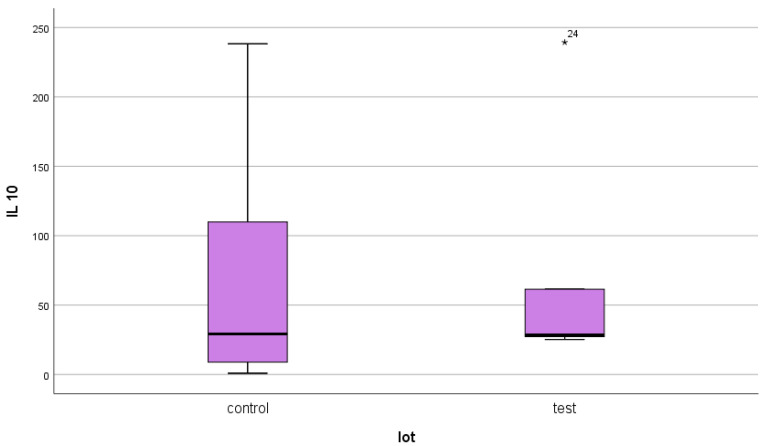
Box and whisker plot for those with non-zero serum IL-10 values (control: non-DM and test: DM). The asterisks represent outlier values.

As in the case of NT-3 and VEGFβ, we found a very high level of inhomogeneity in serum levels for IL-10, which generated a large standard deviation of the mean (Figure 12, Figure 13 and Figure 14). Thus, of the 15 patients in the test group, only 5 (30%) presented non-zero serum values of VEGFβ (meaning values above 1 pg/mL, the detection limit of the ELISA kit used), while of the 48 patients in the control group, 15 had non-zero values (31.25%) (Figure 13 and Figure 15). The rest showed values below the detection limit of the ELISA kit used. It should be noted that the proportion of patients with non-zero (detectable) serum IL-10 levels is similar for both groups.

We analyzed all the results obtained, followed by an aspect that we consider important: how many patients in each group simultaneously presented non-zero serum values for NT-3, VEGFβ, and Il-10.

The results were as follows: from the test group (ESRD + DM), only two patients, both women (39 and 82 years old), presented non-null values for all three parameters. Very interestingly, the 82-year-old patient presented the maximum values obtained in the entire group for NT-3, VEGFβ, and IL-10 [42]. For the control group (ESRD), non-null values for all studied markers were obtained in an 83-year-old female patient. She presented the maximum values of the group for NT-3 and VEGFβ, and for IL-10 an increased, but not maximum, level. Thus, elderly women presented this maximal pattern.

**Figure 16:**
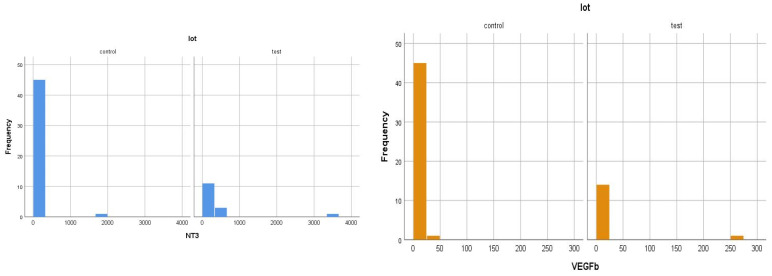

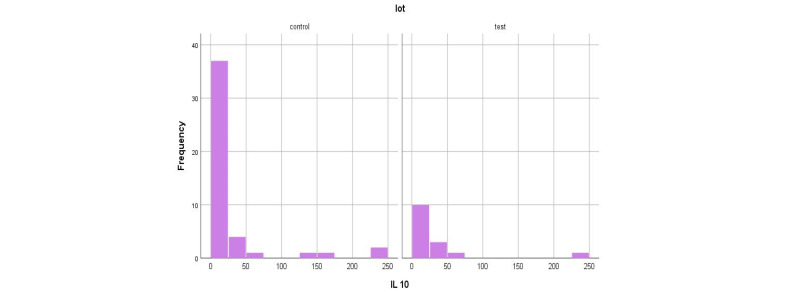
Histogram graphs (data frequency) (control: non-DM and test: DM). These graphs illustrate that the majority of the results obtained are within the 0.00 level range or slightly above.

In Figure 16 and Table 2 and Table 3 we have tried to systematize all the important results obtained in this study.

Preliminary conclusion: most patients in the two groups presented low serum levels for the three studied parameters. We must try to explain the mechanisms by which these two categories of functional behavior are delimited. We must also try to find a possible way to explain the functional interference between NT-3, VEGFβ, and Il-10.

## 5. Discussions

NT-3. After consulting the specialized literature, we can state that this study is the first conducted on the serum behavior of NT-3 in both ESRD + DM patients and those with ESRD only. The determinations performed in the two groups showed some paradoxical changes that allowed us to state that in these categories of patients there is an obvious polarization of the serum pattern of NT-3.

Although the test group (ESRD + DM) had a mean serum NT-3 level 7.8 times higher than the control group (ESRD) and 15.26 times higher than the upper limit of normal, only 40% of the test group patients had non-null serum values. The remaining 60% showed values below the detection limit of the ELISA kit used.

In the control group, although the mean serum NT-3 value was only 2.28 times higher than the upper limit of normal, only 8% of patients presented non-zero levels. We again note an extreme polarization: 92% of the patients in the control group had NT-3 levels below the detection limit of the kit.

Before starting an analysis of the mechanisms that justifies this behavior of NT-3, we issue some preliminary conclusions:

1. most of the patients included in the study (60% of the test group and 92% of the control group) have very low serum NT-3 values (<4 pg/mL);
2. only 40% of the patients in the test group and 8% of those in the control group had such high serum levels of NT-3 that they determined the average values to be so high, compared to normal levels;
3. DM is a pathology that generates additional increases in serum NT-3 only in some of the patients, compared to ESRD.

NT-3 belongs to the neurotrophin family, along with NGF (nerve growth factor), BDNF (brain-derived neurotrophic factor), and NT-4 (neurotrophin-4). All of these factors share common ancestral genes and structural similarities [43]. Classically, NT-3 is known to stimulate the genesis and survival of neurons in both the central and peripheral nervous systems. More recently, it has been shown that NT-3 also modulates synaptic transmission [44]. It is the only neurotrophin capable of activating all tyrosine kinase receptors used by neurotrophins (TrkA, TrkB, and TrkC). It can also bind to the p75NTR receptor (belonging to the tumor necrosis receptor family), which is the first receptor discovered to be used by NT-3. NT-3 has a low affinity for this receptor and can act as its co-receptor. The highest binding affinity of NT-3 is for the TrkC receptor, which is only activated by NT-3 [43,44,45].

In addition to its role in stimulating neuronal survival, differentiation, and regeneration, NT-3 also has angiogenic action, both under normal conditions and in ischemic tissues (reparative neoangiogenesis) [46]. This role is exerted predominantly by the stimulation of TrkC receptors, followed by activation of the PI-3K-Akt-eNOS pathway. Another important aspect proven is that NT-3 does not simultaneously participate with VEGFα in neovascularization [46].

As such, a growth factor initially considered as only neuroregenerative in fact acts in a complex way, stimulating both neuronal and vascular regeneration.

Another action of NT-3 is to stimulate the growth of some parenchymal cells. Thus, NT-3 secreted by hepatic stellate cells is discharged into the small space between them and hepatocytes. The result is the activation of hepatocyte TrkB receptors, with cell proliferation in the middle lobular regions [47].

Increased levels of NT-3 and TrkC receptors are also proven to be found at the renal level, without their actions being clearly known [48]. However, it is certain that NT-3 stimulates the development and survival of neuronal precursors in the human embryonic kidney and inhibits bicarbonate reabsorption in the ascending limb of the loop of Henle in the adult mouse kidney [49,50].

It is essential to mention that, apart from neurons, podocytes are among the few cell types that present TrkC receptors, and therefore one of the few on which NT-3 acts [51]. On the other hand, studies on mouse nephrons have shown that TrkC is more abundant in glomeruli than in tubular cells and is essential for proper glomerular function [51]. Combining these data, we can conclude that NT-3 actually controls proper glomerular function through the podocytes that coat the glomerular capillary wall.

But what happens in ESRD?

From a histological point of view, the characteristic changes consist in diffuse glomerulosclerosis and vascular sclerosis, as well as severe tubulo-interstitial fibrosis that induces tubular atrophy. Added to these aspects is the filling of the tubules with proteinaceous casts that dilate them and chronic inflammation [52]. As an effect of these changes, the disappearance or extreme reduction in the number of podocyte TrkC receptors on which NT-3 could act at the renal level is clear. These histological changes are certainly present in patients from both groups studied.

The very low serum values, below the detection limit of the kit used (<4 pg/mL), in 60% of the patients in the test group and 92% of those in the control group may have the following meanings:

- very low overall capacity for neuroregeneration, neuronal survival, and functional control of synapses, even though these are essential in conditions of diabetic neuropathy;
- reduced or inhibited angiogenic capacity through NT-3 antagonists, even in the presence of diabetic arteriopathy;
- the canceling of kidney production of NT-3;
- the existence of an intense inflammatory background in patients in the studied groups, with very high serum levels of IL-6 [28]. It is already proven that, in high concentrations, IL-6 inhibits BDNF synthesis [53]. We can state that our study confirms that chronic inflammation, namely, high serum levels of IL-6, also inhibits NT-3 secretion.

However, 40% of the patients in the test group and 8% of those in the control group showed very high serum NT-3 values (polarized behavior). What could be the explanation?

Due to the severe renal histological changes in ESRD, amplified by DM, we can hypothesize that the increased serum NT-3 levels do not originate from the kidneys.

We consider that the sources of NT-3 are ischemic tissues and chronic inflammatory foci (caused by uremia and dialysis), rather than the kidneys. It should be remembered that all patients included in the study did not present acute ischemic and/or inflammatory syndrome at the time of blood sample collection.

In addition, it is important to remember that the patients with the maximum NT-3 values in the two groups are elderly and very close in age (82 and 83 years, respectively), so it is very likely that NT-3 is secreted from degenerative nervous and cardiovascular tissue changes.

We can conclude that this polarization of serum NT-3 values in the two groups involves the following aspects:

- ESRD generates a decrease in serum NT-3 levels, the main explanation of which is the severe decrease in renal synthesis at the level of nerve endings and podocytes, through specific histological alterations;
- even if DM is associated, diabetic neuropathy and angiopathy can only counteract the renal influence on serum NT-3 levels in 40% of the patients in the test group;
- the very high serum levels presented by a small proportion of patients in both groups indicate chronic extrarenal production of NT-3, most likely from degenerated nervous and cardiovascular structures. However, it is possible that these levels precede the onset of acute ischemia in different areas of the body. We allow ourselves to advance this idea because the 82-year-old patient in the test group who presented the maximum serum values of the three markers [42] passed away a few months after the end of our study. Relatives could not be contacted, but dialysis colleagues had information about a fatal stroke.

VEGFβ presented a much higher average concentration in the studied groups compared to the upper limit of the internationally accepted normal level (36 times higher in the test group and 2 times higher in the control group). Moreover, the average level in the test group was 17 times higher than in the control group (*p* = 0.04). Paradoxically, however, only 13.33% of the patients in the test group (ESRD + DM) and 8.33% of those in the control group (ESRD) presented non-zero serum VEGFβ values. However, it is important to reiterate that, for VEGFβ, the detection limit of the ELISA kit used is close to the internationally accepted upper limit of normal (<0.4 ng/mL kit limit and 0.467 ng/mL upper average limit of normal). Therefore, we consider that all studied patients who presented zero serum levels of VEGFβ (86.67% of the test group and 91.67% of the control group) actually have normal or even low values. One of the objectives of this study is to understand this apparently paradoxical behavior of VEGFβ.

Due to structural and receptor signaling homology with VEGFα (47% of its amino acid sequence), VEGFβ has long been considered to have an angiogenic role [54,55,56,57].

The VEGFRb signaling pathway involves its binding to the VEGFR-1 (tyrosin kinase receptor present in the heart, kidney, liver, and brain [58]) and the co-receptor NP-1 (neuropilin-1) [54]. VEGFβ binds to the same locus of VEGFR-1 as VEGFα, but with higher affinity. Although the binding affinity of VEGFβ is high, the tyrosine kinase activity of VEGFR-1 is reduced by the existence of a juxtamembrane inhibitory sequence [59,60]. This explains the reduced angiogenic activity of VEGFβ [54]. VEGFR-1 has a very important role in podocyte function and the structural and functional maintenance of the glomerular membrane. It is proven that there are two isoforms of VEGFR-1, one transmembrane and the other soluble, sVEGFR-1/sFLT-1, capable of binding all VEGF subtypes. It is also proven that pericytes from many tissues, including glomerular ones (podocytes), produce sFLT-1, which binds to the glycosphingolipid GM3 in the lipid raft structure of the glomerular endothelial cell membrane. In this way, sFLT-1 controls the normal functioning of the cytoskeleton that articulates on the inner side of the membrane [61]. Deletion of the sFTL-1 gene induces an aberrant reorganization of the cytoskeleton, leading to the appearance of massive proteinuria and renal failure [61].

Thus, although it is present in most tissues and organs, VEGFβ deficiency does not generate a decrease in angiogenesis in any of them. Furthermore, studies have shown that it plays a minor role in angiogenesis, both under normal and pathological conditions [62]. Also, VEGFβ does not cause increased vascular permeability, being the only member of the VEGF family that does not have this role. However, it has been proven that VEGFβ stimulates the revascularization of ischemic myocardium, without knowing whether it exerts a direct or indirect action [62,63]. Another proven aspect is the stimulation of antiapoptotic genes in myocardocytes, cells on which it exerts a direct stimulation of survival [64]. Subsequently, this stimulation of survival was also proven in vascular cells in the cornea and retina [65].

Thus, VEGFb does not induce angiogenesis, but it does induce the survival of many types of cells: vascular (endothelial, pericytes, and smooth muscle cells), neurons (cortical, retinal, and from the spinal cord), and myocytes [66,67,68,69].

A very important aspect to mention is that, in patients with DM, it has been shown that a significant increase in serum VEGFβ levels is associated with diabetic kidney disease (DKD) [70]. Our study also confirmed this, with the average value obtained being 36 times higher than the upper limit of normal. However, as mentioned, the vast majority of patients in both groups had normal or low serum VEGFβ levels.

Another mechanism by which elevated VEGFβ levels accelerate the onset of DKD and progression to ESRD is the stimulation of lipid deposition in glomerular podocytes [71]. This mechanism involves increasing the levels of VEGFβ and VEGFR-1 (inclusive sFLT-1), with the exacerbation of signaling through this pathway.

The use of neutralizing anti-VEGFβ and anti-IL-17 antibodies in mice with DKD generated a decrease in the inflammatory reaction and renal fibrosis, as well as in renal lipid deposits, predominantly triglycerides [72].

In most of the patients included in our study, although ESRD was established, the serum level of VEGFβ was normal or low. However, the paradoxical behavior of the growth factor in 13.33% of the patients in the test group caused the average serum level to exceed the upper limit of normal by 36 times. In the control group, only 8.33% of patients with non-zero VEGFβ values induced increases in the mean level to two times above the upper limit of normal. None of the dialysis patients in the two groups received inhibitory therapy.

Is it possible that the normal or low levels of most ESRD ± DM patients represent a way of inhibiting angiogenesis and/or cellular resistance in some organs that no longer function?

The antiangiogenic nature of VEGFβ has been proven by the fact that it inhibits signaling through the FGF2 (also known as basic fibroblastic growth factor)/FGFR1 (receptor with intracytosolic tyrosine kinase domain) pathway. FGF2 acts on endothelial cells, stimulating angiogenesis, but also their proliferation and migration [73].

At this point, some preliminary hypothetical conclusions can be made that need to be confirmed experimentally (studies conducted on VEGFβ in these categories of patients are very few). Thus, in patients with elevated VEGFβ values in both groups, we can consider the following causes:

- there is still active renal tissue with inflammatory reaction, fibrosis, and eventual angiogenesis;
- there is DM-induced angiopathy, which the body fights against through the hyperproduction of VEGFβ, with an antiapoptotic action on damaged vessels;
- there are associated cardiovascular and neurodegenerative pathologies in which the affected tissues require the antiapoptotic and proregenerative actions of VEGFβ;
- in both pathologies, there is an intense inflammatory background proven by our previous studies [28,42]. Under these conditions, IL-1β, TNFα, and IL-17 stimulate the VEGFβ synthesis from activated macrophages. But the same macrophages also secrete IL-10, an anti-inflammatory cytokine, which inhibits the VEGF synthesis [74].

IL-10 presented similar mean serum values in both groups, at approximately double the upper limit of normal (2.6 times higher in the test group and 2.2 times higher in the control group). Also, the maximum values were very close, over 20 times higher than the upper limit of normal.

From the beginning, we can conclude that DM does not induce a significant change in IL-10 serum levels.

Another interesting aspect is the maintenance of the same serum IL-10 behavior polarization as for the growth factors studied. Thus, only 30% of the patients in the test group and 31.25% of those in the control group presented serum values above the detection limit of the kit used (>1 pg/mL). However, their serum IL-10 levels were so high that they doubled the average value.

Among the patients in the test group with elevated serum NT-3, 66.7% also had elevated IL-10. All patients in the test group with elevated serum VEGFβ also had elevated IL-10.

In the control group, all patients with increased NT-3 also presented increased serum values for IL-10, while only one patient with increased VEGFβ also presented increased IL-10.

How can these results be understood?

The involvement of IL-10 in renal pathology is clearly proven. Data from the literature describe an increase in serum IL-10 levels in ESRD as a way of defending against multiple inflammatory reactions induced in the body by uremia and chronic dialysis [75].

However, things are not so unidirectional.

IL-10 is secreted by T and B cells, NK cells, monocytes, macrophages, and dendritic cells. It binds to specific receptors (IL-10Rα/1 and IL-10Rβ/2), after which it generates the activation of the kinases Jak1 (Janus kinase 1) and Tyk2 (tyrosine kinase 2). Activated kinases induce the activation of transcription factors STAT1, STAT3, and STAT5, inhibiting the inflammatory reaction and triggering immune responses [76]. By stimulating the leukocyte receptors IL-10Rα/1, IL-10 induces a decrease in the secretion of pro-inflammatory cytokines (IL-1β, TNFα, IL-12, and others). IL-10 has an important role in modulating the fibrosis that occurs at the end of the inflammatory reaction, with dual effects being proven, both stimulatory and inhibitory [77,78].

At the renal level, the main source of IL-10 is represented by mesangial cells, which form the renal mesangium together with the matrix [79,80].

Under normal conditions, these cells control renal function: they secrete the extracellular matrix that supports the glomeruli, modulate the glomerular filtration rate by controlling the size of the capillary loops, and represent a source and target for hormones and growth factors [81,82,83].

Mesangial cells exhibit different morphologies, depending on their level of activation. In the inactive form, they do not show increased protein synthesis, have a stellate shape, a low mitotic rate, and sparse cytoplasm. In the activated state, the cell acquires an elongated shape, has an increased rate of mitosis, and massively synthesizes alpha smooth muscle actin (α-sma) and interstitial collagen. This activated form is present in renal pathology [84,85,86].

In the activated/proliferative form, mesangial cells begin to secrete large, excessive amounts of cytokines (including IL-10), vasoactive hormones, growth factors, and mesangial matrix proteins. It is proven that these secretion products stimulate in an autocrine manner the mesangial cells that produced them [87,88]. Under these conditions, IL-10 acts as a growth factor, inducing the appearance and maintenance of their activated/proliferative form.

The secreted factors also generate the paracrine transmission of information to endothelial cells, tubular nephrocytes, and leukocytes that have infiltrated the renal parenchyma [87,88].

How is this transmission accomplished?

The mesangial cell is considered to be similar to myofibroblasts present in other tissues. The most important structural characteristic is that it has cytoplasmic extensions/processes that extend from the cell center towards the capillary lumen. In these extensions are fibrils with diameters of 7–10 nm, through which the mesangial cell is connected to the surrounding matrix and to the glomerular basement membrane, regarding the endothelial cells [89]. Thus, they connect to the glomerular basement membrane both directly and through the mesangial matrix.

Mesangial expansion plays an important role in the development of diabetic nephropathy and involves an excessive proliferation of mesangial cells and the deposition of matrix proteins in the center of the glomerulus [90]. However, the mechanisms by which mesangial expansion causes renal failure are not clarified.

The mesangial matrix contains low-density type IV collagen, proteoglycans (including heparan sulfate), fibronectin (large amounts), and some laminin [89]. On the other hand, the glomerular basement membrane (GBM) with which the matrix is connected contains high-density type IV collagen, heparan sulfate, and laminin [91].

These structural differences between the GBM and mesangial matrix also generate functional differences. Thus, low-density type IV collagen together with hydrophilic proteoglycans allow the mesangial matrix to resist both expansion and compression. The high-density type IV collagen in the GBM allows resistance to increased blood pressure in the glomerular capillaries [89]. In diabetic nephropathy, part of the type IV collagen is replaced by type III collagen [92].

Mesangial cells also have a role in consolidating the GBM, which is discontinuous. A portion of the capillary wall is directly exposed to the mesangium, consisting only of mesangial cells. The endothelial cell layer is very thin and strongly fenestrated [93]. Under these conditions, mesangial cells anchor the GBM in order to withstand the increased blood pressure inside the glomerular capillary. When the patient has hypertension, a common feature in DM, the blood in the glomerular capillaries exerts increased pressure on the GBM, with the breaking of connections between the GBM and the mesangium and eventual expansion of the glomerular capillaries [94]. Therefore, increased hydrostatic pressure in the glomerular capillaries can cause the rupture of GBM–mesangial cell connections, with a consequent expansion of the mesangium [94,95].

From all of the above, it appears that IL-10 exerts a dual role: on the one hand, it acts as a powerful anti-inflammatory and immune stimulator, on the other hand, it is a powerful activator of fibrosis and structural and functional renal degeneration.

It should not be overlooked that patients in both groups tested present strong inflammatory backgrounds [28], that the inflammatory foci have different stages of evolution, and that this is the real background on which IL-10 must act.

Under these conditions, we cannot state with certainty whether the polarized aspect of the obtained serum IL-10 levels exerts a strictly negative or a strictly positive effect.

The high and very high concentrations obtained in approximately 30% of the patients of each group studied denote both the existence of a general anti-inflammatory background that tries to oppose the strong inflammation induced by uremia, dialysis, and other associated pathologies, as well as an accentuated profibrotic tendency both at the renal level and in the other inflamed tissues.

However, the remaining patients (approx. 70%) from both groups studied, who presented serum IL-10 values below the detection limit of the Merck Millipore (Romania office) ELISA kit used (<1 pg/mL), have a very poor ability to counteract the strong inflammatory background existing in each of them.

### Functional Interrelationships

- We cannot state that there is a mutual stimulation mechanism between NT-3 and VEGFβ.

The arguments for this are as follows:

- Of the fifteen patients in the test group, only two (13.33%) showed elevated values for both NT-3 and VEGFβ.
- In the control group (forty-five patients), only one (2.22%) showed this aspect.

- Can we talk about a negative influence that each of the two growth factors exerts on the other?

The argument in favor of this is that four of the six patients in the test group (26.66% of the total) with non-null NT-3 values had associated normal or low VEGFβ.

The argument against this is that three of the four patients in the control group (9.99% of the total) with non-zero NT-3 values had associated non-zero VEGFβ.

In any case, each of the two growth factors signals intracellularly through tyrosine kinase receptors, and substrate competition may occur between the cytosolic kinases involved in the two pathways. However, situations are also described in which the generation of signals through one type of receptor tyrosine kinase pathway also activates another tyrosine kinase pathway, associated with another type of receptor [96].

- In both groups’ patients with maximum values of NT-3 and/or VEGFβ, well above the non-null values of the others, can a structural alteration of the receptors be considered as the cause? That is, is it possible that the huge values are induced by the inability to signal through these receptors? Is it possible that the intracellular signaling of NT-3 and/or VEGFβ is extremely reduced despite the very high serum values?
- IL-10 stimulates the synthesis of NT-3, especially in adult neural stem cells. In this way, IL-10 indirectly stimulates the synthesis of neurons, oligodendrocytes, axonal growth, and myelin repair [97]. We can confirm this aspect based on the results obtained in the test group. Of the patients with normal or elevated NT-3 values, 83.33% also showed elevated IL-10 values. All patients (100%) in the control group with normal or elevated NT-3 values showed elevated IL-10 values. We can state that the presence of DM slightly decreases the positive correlation between serum NT-3 and IL-10 values.
- Regarding the scientific data describing the functional interrelationship between IL-10 and VEGFβ, the information is very limited. The existing works consulted describe either the interrelationship between IL-10 and total VEGF, or that between IL-10 and VEGFα. In the patients we studied, we found that all those in the test group (ESRD + DM) with elevated VEGFβ values had associated elevated serum levels of IL-10 (100%). Of the patients in the test group who showed elevated VEGFβ levels, 75% also had elevated IL-10 levels.
- Although the anti-inflammatory, anti-atherosclerotic, and antithrombotic effects of IL-10 are known, a prospective study suggested that elevated serum levels of IL-10 are associated with an increased risk of cardiovascular events in patients with CKD [98]. Under these conditions, the simultaneous presence of elevated serum levels of NT-3, VEGFβ, and IL-10 in patients from both groups could be considered a very important prognostic marker for acute cardiac and vascular events, including at the level of the central and peripheral nervous system.

## 6. Conclusions

1. Significantly increased mean serum levels were obtained for the two growth factors (NT-3 and VEGFβ) in the test group patients (ESRD + DM) compared to those in the control group (ESRD only).
2. Compared to the internationally accepted upper limits of normal, the mean serum levels of NT-3 and VEGFβ were much higher.
3. The major polarization of serum NT-3 and VEGFβ values was very surprising. Although most patients had very low serum levels (most frequently below the detection limit of the kits), some of them had huge levels, which generated an elevated mean value.
4. This polarization cannot be attributed to the quality of the kits or the conditions of collection, preservation, and processing of biological samples. The results presented in this article belong to a very wide range of studied parameters. Although kits of the same origin were used for other markers, and the dosages were performed on the same day for all the markers studied, polarizations were present only for NT-3 and VEGFβ, and to a lesser extent for IL-10.
5. In patients of both groups with very high NT-3 and VEGFβ values, we consider that their source is most likely extrarenal, from areas affected by diabetic neuropathy and angiopathy but also from the multiple inflammatory foci generated in the body by chronic uremia and hemodialysis. This does not exclude the possibility of low renal production.
6. The very low levels of NT-3 in most of the studied patients indicate a severe decrease in its renal production at the level of nerve endings and podocytes. We believe that the kidney should be reconsidered as an important source of this growth factor.
7. Very low VEGFβ values, below the detection limit of the kit which is very close to the upper limit of normal serum levels, actually mean normal or low values. It is possible that these values obtained in most patients in the two groups signify an inhibition of angiogenesis induced by ESRD, as well as an intense proapoptotic tendency of the cells.
8. IL-10 presented similar mean serum values in the two groups at approximately double the upper limit of normal. This aspect leads us to conclude that, on the one hand, DM does not have a major influence on the serum level of IL-10, and on the other hand, even if this anti-inflammatory cytokine has elevated values, it cannot counteract the pronounced inflammatory background existing in both categories of patients.
9. We can discuss the existence of a negative/inhibitory influence that each of the two growth factors exerts on the other, predominantly on the signaling pathway. Each of the two growth factors utilizes cytosolic tyrosine kinases, between which substrate competition can occur.
10. In patients with maximum NT-3 and VEGFβ values, we can consider both the existence of an imminent ischemic-neurodegenerative event, as well as the structural and functional alteration of the specific receptor (the decrease/absence of intracellular signaling triggers positive feedback on the ligand synthesis and release into the serum).
11. Positive correlations were obtained between NT-3 and IL-10, as well as between VEGFβ and IL-10. Especially for the latter, the information is extremely limited, so these correlations may represent an unexplored area of research.
12. IL-10 is not only an anti-inflammatory, anti-atherosclerotic, and antithrombotic cytokine, but also a profibrotic one. Furthermore, experimental data have shown that elevated serum levels of IL-10 are associated with an increased risk of cardiovascular events in patients with KCD.
13. Under these conditions, the simultaneous presence of elevated serum levels of NT-3, VEGFβ, and IL-10 could announce the imminence of acute cardiac and/or neurovascular events. It is certainly a phenomenon that requires very careful further research. If this aspect is confirmed, it may be a very important predictive marker that requires the introduction of intensive preventive therapy.

## Figures and Tables

**Table 1:** General information about patients included in the study.

Hemodialysis Patients Included in the Study	Average Age	Sex	CKD Duration	Hemodialysis Duration
Non-DM (45)	61.5 ± 15.1 yrs.	33 ♂, 12 ♀	1–24 yrs.	5.29 ± 4.71 yrs.
DM (15)	63 ± 11.8 yrs.	9 ♂, 6 ♀	1–14 yrs.	2.53 ± 2.69 yrs.

**Table 2:** Descriptive statistics of the parameters in the studied groups (non-zero values were eliminated).

Reduced Group		NT-3	VEGFβ	IL-10
Control (ESRD)	Numer of Patients	4	4	15
	Minimum	43.00	0.78	0.89
	Maximum	1718.71	41.65	238.30
	Mean	525.49	11.75	67.45
	Std. Deviation	797.76	19.94	85.00
	Median	170.14	2.30	29.16
Test (DM + ESRD)	Number of Patients	6	2	5
	Minimum	95.86	4.69	25.23
	Maximum	3594.43	251.21	239.32
	Mean	888.09	127.95	76.35
	Std. Deviation	1335.95	174.31	92.31
	Median	396.57	127.95	28.5
Total	Number of Patients	10	6	20
	Minimum	43.00	0.78	0.89
	Maximum	3594.43	251.21	239.32
	Mean	743.0560	50.4883	69.6815
	Std. Deviation	1112.98942	99.5795	84.4646
	Median	308.0000	3.6050	28.8450

**Table 3:** Systematization of the important results obtained in the two groups.

Measured Marker	Mean Serum Level and Standard Deviation	Maximum Value, Age, Gender	Minimum Value, Age, Gender	Number of Patients with Non-Zero Level	Number of Patients with Zero Level
**Test group (DM + ESRD)** **NT-3**	354.24 ± 916.64 pg/mL	3594.43 pg/mL 82 yrs., ♀	0 (<4 pg/mL) 50–83 yrs., 6 ♂, 9 ♀	6	9
**Control group (ESRD) NT-3**	45.7 ± 254.64 pg/mL	1718.71 pg/mL 83 yrs., ♀	0 (<4 pg/mL) 28–88 yrs., 30 ♂, 12 ♀	4	41
**Test group (DM + ESRD)** **VEGFβ**	17.06 ± 64.8 ng/mL	251.21 ng/mL 82 yrs., ♀	0 (<0.4 ng/mL) 50–83 yrs., 7 ♂, 5 ♀	2	13
**Control group (ESRD) VEGFβ**	1.02 ± 6.14 ng/mL	41.65 ng/mL 83 yrs., ♀	0 (<0.4 ng/mL) 28–88 yrs., 30 ♂, 11 ♀	4	41
**Test group (DM + ESRD)** **IL-10**	25.45 ± 61.83 pg/mL	239.32 pg/mL 82 yrs., ♀	0 (<1 pg/mL) 50–83 yrs., 7 ♂, 3 ♀	5	10
**Control group (ESRD)** **IL-10**	21.99 ± 57.18 pg/mL	238.3 pg/mL 52 yrs., ♂	0 (<1 pg/mL), 28–88 yrs., 9 ♀, 22 ♂	14	31

## Data Availability

The authors consent to data sharing in a publicly accessible repository.
